# Supplementary material for: Mono- to tetra-alkyl ether cardiolipins in a mesophilic, sulfate-reducing bacterium identified by UHPLC-HRMSn: a novel class of membrane lipids
Source: Front Microbiol. 2024 May 22;15:1404328. doi: 10.3389/fmicb.2024.1404328 (PMC11150832; doi:10.3389/fmicb.2024.1404328)
Supplement: Supplementary file 1 [file Data_Sheet_1.PDF]

Supplementary Figures and Tables

Mono- to tetra-alkyl ether cardiolipins in a mesophilic, sulfate-reducing bacterium identified by UHPLC-HRMS^n: A novel class of membrane lipids

Ellen C. Hopmans[a‡], Vincent Grossi[b#‡], Diana X. Sahonero Canavesi[a], Nicole J. Bale[a], Cristiana Cravo-Laureau[c], Jaap S. Sinninghe Damsté[a,d*]

[a] *NIOZ Netherlands Institute for Sea Research, Department of Marine Microbiology and Biogeochemistry, PO Box 1790 AB 59, Den Burg, The Netherlands*

[b] *Univ Lyon, UCBL, Laboratoire de Géologie de Lyon: Terre, Planètes, Environnement (LGL-TPE, UMR CNRS 5276), Villeurbanne, France*

[c] *Université de Pau et des Pays de l'Adour, E2S UPPA, CNRS, IPREM, Pau, France*

[d] *Utrecht University, Faculty of Geosciences, Department of Earth Sciences, P.O. Box 80.021, 3508 TA Utrecht, The Netherlands*

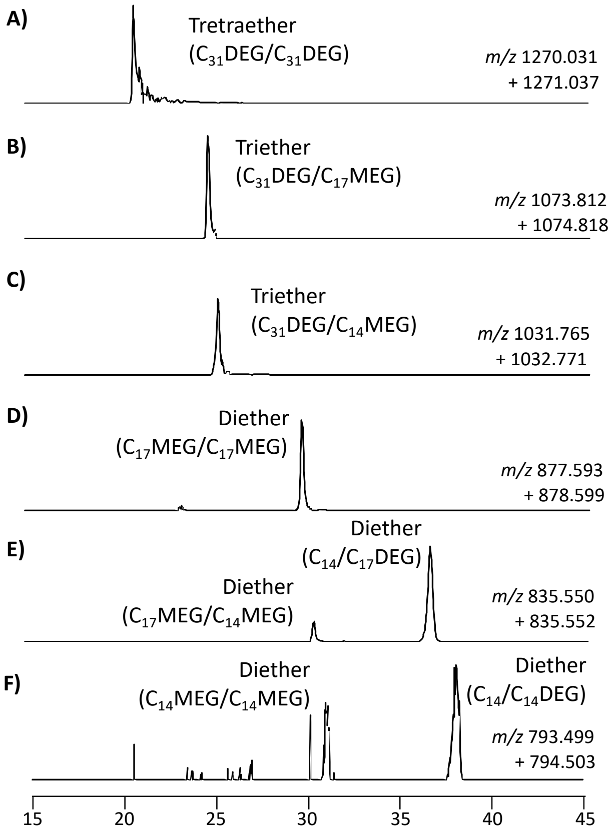

Figure S1. Partial mass chromatograms of cardiolipins and lyso-cardiolipins with ether-bound alkyl chains. **A)** tetraether cardiolipin with 62 alkyl carbon atoms composed of two C_{31}DEG with C_{17} and C_{14} alkyl chains; **B)** monolyso-cardiolipin with 48 alkyl carbon atoms composed of C_{14}/C_{17}DEG + C_{17}MEG (peak **n**); **C)** monolyso-cardiolipin with 45 alkyl carbon atoms composed of C_{14}/C_{17}DEG + C_{14}MEG; **D)** dilyso-cardiolipin with 34 alkyl carbon atoms composed of two C_{17}MEG (C_{17}/C_{17}DEG was not detected); **E)** dilyso-cardiolipins with 31 alkyl carbon atoms composed of C_{14}MEG+C_{17}MEG or C_{14}/C_{17}DEG; **F)** dilyso-cardiolipins with 28 alkyl carbon atoms composed of two C_{14}MEG or C_{14}/C_{14}DEG (peak **p2**). Diagnostic spectra of **C** and **E** are shown in Figure 8.

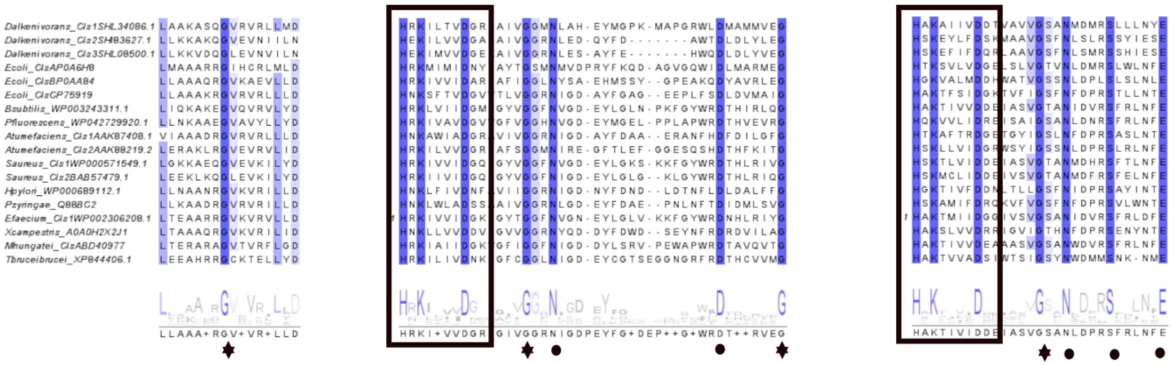

Figure S2. Multiple sequence alignment of the three potential cardiolipin synthases Cls from *D. alkenivorans* and the bacterial-type Cls belonging to the PLD family characterized in bacterial species (*Escherichia coli, Staphylococcus aureus, Enterococcus faecium, Pseudomonas fluorescens, Bacillus subtilis, Agrobacterium tumefaciens, Helicobacter pylori, Pseudomonas syringae, Xanthomonas campestris*), archaea (*Methanospirillum hungatei*) and eukarya (*Trypanosoma brucei brucei*). The partial amino acid sequences of the three putative *D. alkenivorans* Cls containing the two conserved HXKXXXXD motifs (black boxes), the conserved glycines (black stars) and other strictly conserved residues D, N, S and E (black circles) as all other Cls sequences. The amino acid consensus is colored according to 50% identity conservation. The consensus sequence logo is shown under both alignments. Three blocks with the conserved sequence are shown.

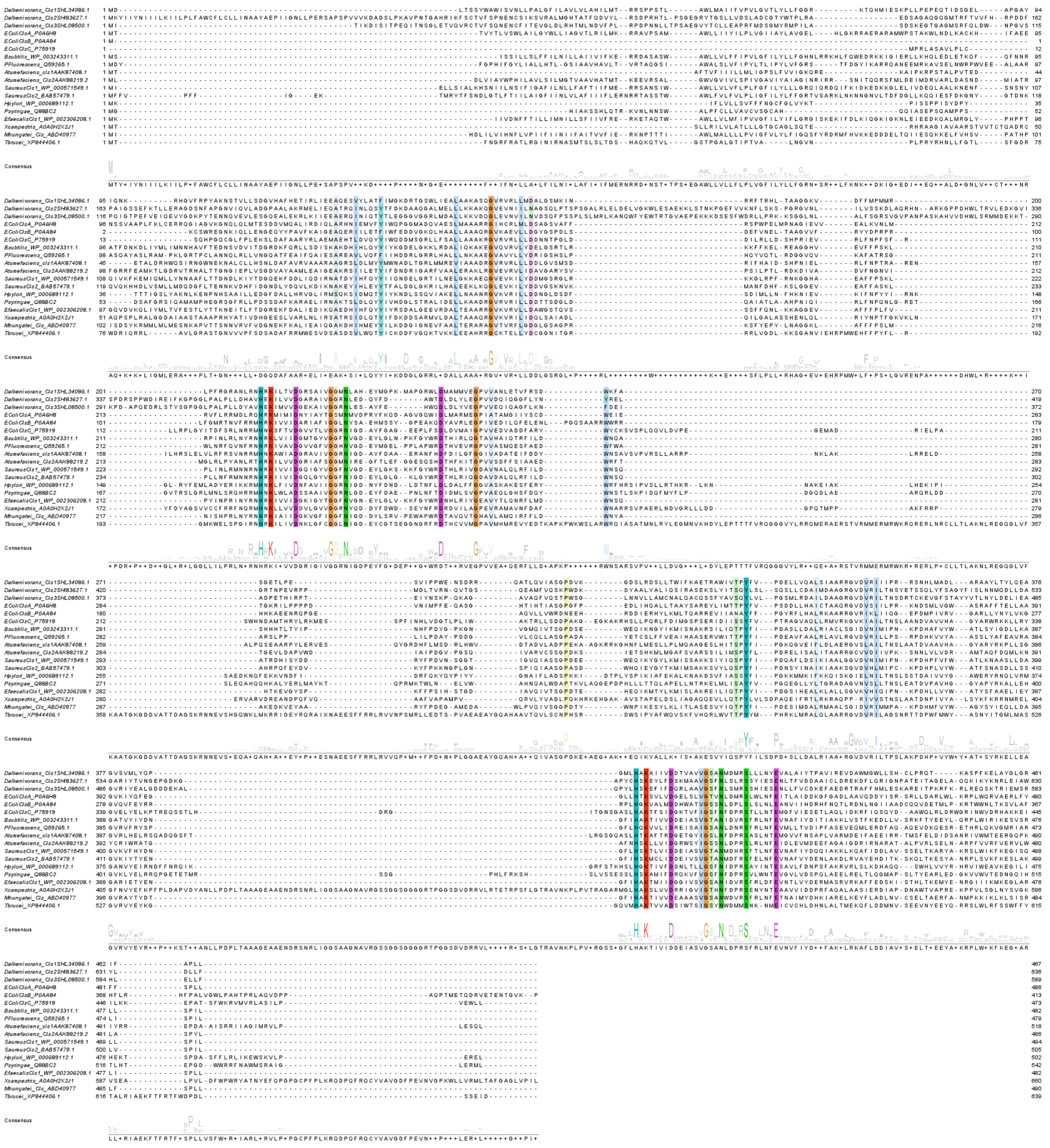

Figure S3. Multiple sequence alignment of the three potential cardiolipin synthases Cls from *D. alkenivorans* and the bacterial-type Cls belonging to the PLD family characterized in bacterial species (*Escherichia coli, Staphylococcus aureus, Enterococcus faecium, Pseudomonas fluorescens, Bacillus subtilis, Agrobacterium tumefaciens, Helicobacter pylori, Pseudomonas syringae, Xanthomonas campestris*), archaea (*Methanospirillum hungatei*) and found in eukaryotes (*Trypanosoma brucei brucei*). Complete amino acid sequences showing the conserved regions along all the cardiolipin synthases (characterized and potential). The residues are colored according to 50% identity conservation. The consensus sequence logo is shown under the alignments.

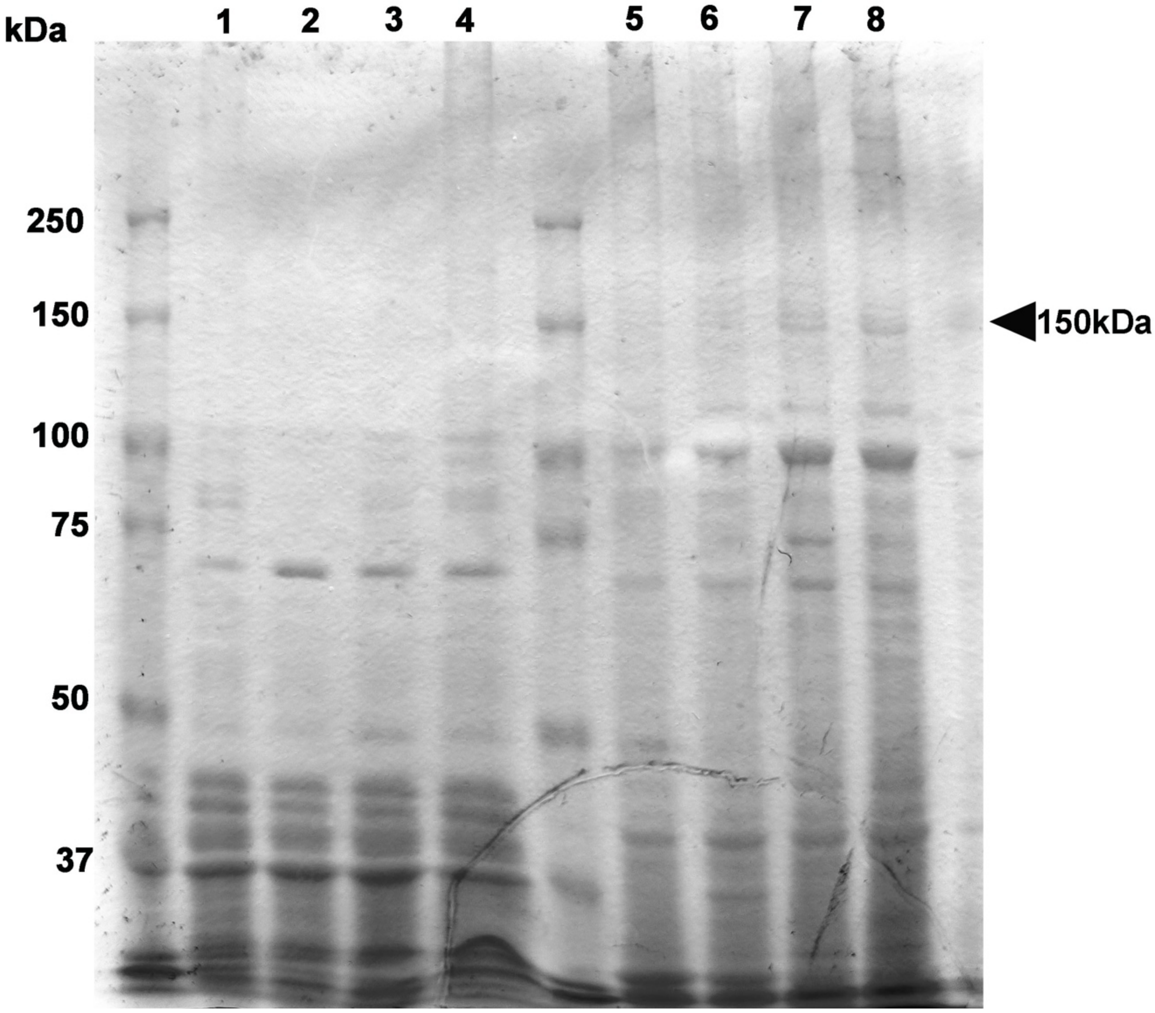

Figure S4. SDS-PAGE gel image of the induced expression in *E. coli* of the Ger2 and Ger proteins of *D. alkenivorans*. The molecular marker is shown in the first and middle lane. *E. coli* BL21 protein extracts carrying the pCDF Duet-1 and pET29b empty vectors (lanes 1 and 5, and 3 and 7, respectively), the Ger2 (SHK01260.1, lane 2 and 6), and the Ger (SHJ90043.1; lane 4 and 8) proteins. Lanes 1-4 correspond to the protein extracts obtained from the soluble fraction and lanes 5-8 to the insoluble fraction (inclusion bodies) after 16 h postinduction. The arrow indicates approx. 150 kDa size of expected proteins. Cells were lysed using B-PERTM bacterial protein extraction reagent and inclusion bodies were prepared according to the manufacturer's mini-scale protocol.

Table S1. Potential genes and associated enzymes involved in the synthesis of bacterial alkyl ether CDL and their lyso-counterparts encoded by the genome of *D. alkenivorans*.

Gene	Enzyme description	Uniprot accession	NCBI accesion (Ref. Seq.)	Number of transmem-brane domains[a]	Membrane protein
cls1	Cardiolipin synthase 1	SHL34086.1	WP_073479011.1	2	Yes
cls2	Cardiolipin synthase 2	SHI83627.1	WP_139264592.1	1	Yes
cls3	Cardiolipin synthase 3	SHL08500.1	WP_073478579.1	ND	Yes
ymdB	O-acetyl-ADP-ribose deacetylase	SHL16402.1	WP_073478717.1		
ger	Glycerol ester reductase	SHJ90043.1	WP_073476268.1	1*	Yes
ger2	Potential Glycerol ester reductase 2	SHK01260.1	WP_073476658.1	ND	Yes
cdsA	phosphatidate cytidylyltransferase	SHI78129.1	WP_073472543.1	6	Yes
pgsA	CDP-diacylglycerol-glycerol-3-phosphate 3-phosphatidyltransferase	SHK52233.1	WP_073477644.1	5	Yes
pgpA	Phosphatidylglycerophosphatase A	SHJ07628.1	WP_073473540.1	3	Yes
pssA	CDP-diacylglycerol-serine O-phosphatidyltransferase	SHL43585.1	WP_083611310.1	6	Yes
psd	phosphatidylserine decarboxylase family protein	SHL43626.1	WP_073479141.1	2	Yes
pla1	phospholipase A1	SHK83058.1	WP_073478171.1	1	Yes
	Patatin-like phospholipase 1	SHJ82754.1	WP_073475982.1		

[a] transmembrane domains were detected by application of the TMHMM algorithm (Krogh et al., 2001)

Table S2. The sequence similarity as determined by a repeated PSI_BLAST searches of ClsA, ClsB, ClsC, archaeal Cls, the bifunctional Cls/PEs and an "eukaryote-like" Cls compared with proteins encoded by the genomes of *D. alkenivorans* and the closely related species *D. aliphaticivorans*. The sequence similarity for Ger and Ger-like is also provided.

Species			*E. coli*		*M. hungatei*	*X. campestris*	*S. coelicolor*	*D. alkenivorans*
Protein		**ClsA**<br>P0A6H8<br>(486 AA)	**ClsB**<br>P0AA84<br>(413 AA)	**ClsC**<br>P75919<br>(473 AA)	**arCls**<br>A0A8F5VN54<br>(504 AA)	**Cls/PEs**<br>WP_011035434.1<br>(660 AA)	**Cls**<br>Sco1389<br>(215 AA)	**Ger2**<br>SHJ90043<br>(1458 AA)
Desulfatibacillum alkenivorans								
**Cls1**	Evalue	0	2E-143	1E-127	0	8E-69		
WP_073479011.1	query cover (%)	88	76	82	93	41		
(467 AA)	similarity (%)	30.9	32.5	25.6	30.2	26.0		
**Cls2**	Evalue	2E-112	3E-74	X6E-86	6E-105	2E-23		
WP_139264592.1	query cover (%)	72	73	65	75	36		
(612 AA)	similarity (%)	25.9	20.9	21.2	26.2	30.5		
**Cls3**	Evalue	9E-86	9E-85	3E-81	1E-117	3E-21		
WP_073478579.1	query cover (%)	66	53	63	57	34		
(589 AA)	similarity (%)	27.3	24.7	23.5	25.0	29.4		
	Evalue						6E-56	
WP_073477644.1	query cover (%)						56	
(195 AA)	similarity (%)						34.4	
**Ger**	Evalue							0
SHK01260	query cover (%)							96
(1458 AA)	similarity (%)							25.9
Desulfatibacillum aliphaticivorans								
**Cls1**	Evalue	0	1E-141	4E-125	0	4E-70		
WP_015948190.1	query cover (%)	88	76	82	93	71		
(467 AA)	similarity (%)	31.3	32.5	25.1	30.4	23.2		
**Cls2**	Evalue	8E-114	5E-76	1E-84	3E-106	2E-24		
WP_136360700.1	query cover (%)	74	73	65	75	37		
(612 AA)	similarity (%)	25.9	21.3	20.6	26.8	30.5		
**Cls3**	Evalue	3E-84	9E-85	5E-83	3E-116	8E-22		
WP_012610398.1	query cover (%)	67	53	63	71	343		
(589 AA)	similarity (%)	27.0	24.7	23.5	25.3	30.6		
	Evalue						4E-56	
WP_012609567.1	query cover (%)						56	
(195 AA)	similarity (%)						35.3	
**Ger**	Evalue							0
WP_028315991.1	query cover (%)							96
(1401 AA)	similarity (%)							25.2
